# pH-dependent delivery of chlorhexidine from PGA grafted mesoporous silica nanoparticles at resin-dentin interface

**DOI:** 10.1186/s12951-021-00788-6

**Published:** 2021-02-09

**Authors:** Zohaib Akram, Sultan Aati, Hein Ngo, Amr Fawzy

**Affiliations:** UWA Dental School, University of Western Australia, 17 Monash Avenue, Nedlands, WA 6009 Australia

**Keywords:** Chlorhexidine, Antimicrobial, Mesoporous silica nanoparticle, pH-sensitive, Drug delivery, Adhesion

## Abstract

**Background:**

A low pH environment is created due to the production of acids by oral biofilms that further leads to the dissolution of hydroxyapatite crystal in the tooth structure significantly altering the equilibrium. Although the overall bacterial counts may not be eradicated from the oral cavity, however, synthesis of engineered anti-bacterial materials are warranted to reduce the pathogenic impact of the oral biofilms. The purpose of this study was to synthesize and characterize chlorhexidine (CHX)-loaded mesoporous silica nanoparticles (MSN) grafted with poly-L-glycolic acid (PGA) and to test the in vitro drug release in various pH environments, cytotoxicity, and antimicrobial capacity. In addition, this study aimed to investigate the delivery of CHX-loaded/MSN-PGA nanoparticles through demineralized dentin tubules and how these nanoparticles interact with tooth dentin after mixing with commercial dentin adhesive for potential clinical application.

**Results:**

Characterization using SEM/TEM and EDX confirmed the synthesis of CHX-loaded/MSN-PGA. An increase in the percentage of drug encapsulation efficiency from 81 to 85% in CHX loaded/MSN and 92–95% in CHX loaded/MSN-PGA proportionately increased with increasing the amount of CHX during the fabrication of nanoparticles. For both time-periods (24 h or 30 days), the relative microbial viability significantly decreased by increasing the CHX content (*P* < 0.001). Generally, the cell viability percentage of DPSCs exposed to MSN-PGA/Blank, CHX-loaded/MSN, and CHX-loaded/MSN-PGA, respectively was > 80% indicating low cytotoxicity profiles of experimental nanoparticles. After 9 months in artificial saliva (pH 7.4), the significantly highest micro-tensile bond strength value was recorded for 25:50 CHX/MSN and 25:50:50 CHX/MSN-PGA. A homogenous and widely distributed 50:50:50 CHX-loaded/MSN-PGA nanoparticles exhibited excellent bonding with the application of commercially available dentin adhesive.

**Conclusions:**

A pH-sensitive CHX release response was noted when loaded in MSN grafted PGA nanoparticles. The formulated drug-loaded nanocarrier demonstrated excellent physicochemical, spectral, and biological characteristics. Showing considerable capacity to penetrate effectively inside dentinal tubules and having high antibacterial efficacy, this system could be potentially used in adhesive and restorative dentistry.
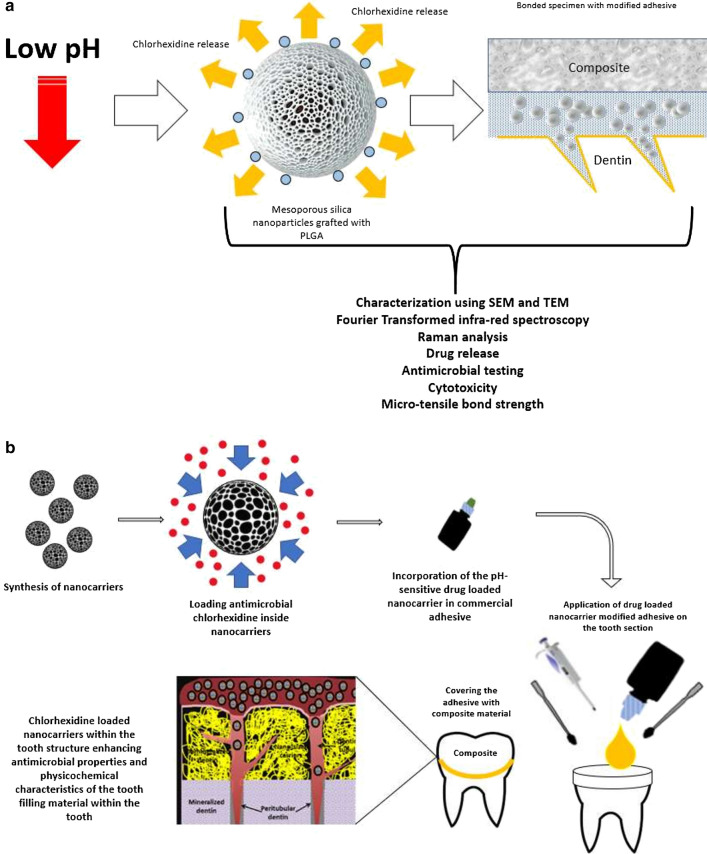

## Background

Effective dentin bonding is by far the most debated topic and gaining more attention in restorative dentistry. The use of contemporary adhesive restorations is widespread, and their long-term clinical success is dependent on their handling characteristics [1, 2]. The resin-dentin interface is the most critical factor and is presumably considered a fragile link between composite and dentin. Two of the most common unwanted failures that are associated with contemporary adhesive restorations that negatively affect clinical success are ‘lack of durability’ and ‘microleakage’ [3].

Bacterial activity at the tooth-restoration interface leads to the development of secondary caries. This is facilitated by the gap formation at the perimeter of the interface that promotes bacterial colonization, thereby facilitating demineralization of the tooth structure [3]. Simultaneously, a low pH environment is created due to the production of acids by biofilms that further leads to the dissolution of hydroxyapatite crystal in the dentin substrate significantly altering the equilibrium [4, 5]. Although the bacterial counts from micro-niche may not be eradicated from the oral cavity, however, synthesis of engineered anti-bacterial materials are warranted to reduce the pathogenic impact of the oral biofilms [6–9].

In recent years, porous materials have offered a promising therapeutic solution to a wide variety of fields [10]. Of these, mesoporous silica nanoparticles (MSNs) are a category of porous materials with exceptional surface area and a significant number of pores that load various quantities of drugs within for improved efficacy [11, 12]. Moreover, these synthesized structures can undergo surface modifications, possess excellent biocompatibility and high pore volume [13, 14]. Numerous research reports have indicated that MSNs can also be modified to be stimuli-responsive controlled release systems, which rapidly release drugs in response to changes in microenvironments of pathological sites or intracellular stimuli [15–18]. Among these stimuli-responsive systems, the pH-responsive system is of particular interest [12]. Poly (L-glycolic acid) (PGA) is another widely studied synthetic polypeptide due to its modifiable carboxyl side group and pH-responsive property [19]. It has been reported that low pH has a significant effect on the drug release behavior and nanoparticle degradation. It is suggested that the drug release from the biodegradable matrix of PGA was matrix-diffusion and polymer-degradation controlled [20, 21]. In some cases, drug properties such as crystallinity and solubility in the release medium also played a critical role in drug release kinetics from PGA [22].

Herein, we reported a facile strategy to graft PGA on the surface of pH-responsive MSN through the N-carboxyanhydride (NCA) ring-opening polymerization of ɤ-benzyl-L-glutamate N-carboxyanhydride (BLG-NCA) [12]. Chlorhexidine (CHX), a biguanide antimicrobial drug was chosen as a model drug to evaluate the drug loading/releasing behavior of MSN-PGA for potential application in adhesive dentistry. We hypothesized that the resultant MSN-PGA would demonstrate superior properties in terms of loading and delivering more CHX compared to the unmodified MSN carrier. The purpose of this study was to synthesize and characterize CHX-loaded/MSN-PGA and to test the in vitro drug release in various pH environments, cytotoxicity, and antimicrobial capacity. In addition, this study aimed to investigate the delivery of CHX-loaded/MSN-PGA nanoparticles through demineralized dentin tubules and how these nanoparticles interact with tooth dentin after mixing with commercial dentin adhesive for potential clinical application.

## Methods

Chlorhexidine base (≥ 99.5%) and other chemicals including _L_-glutamic acid γ-benzyl ester (≥ 99.9%), poly (lactic-co-glycolic acid—PGA; 50/50, mol wt 30,000–60,000), triphosgene reagent grade, 3-aminopropyltriethoxysilane (APS), N-cetyltri-methylammonium bromide (CTAB), tetraethyl orthosilicate (TEOS), phosphate-buffered saline (PBS), MTT assay kit were purchased from Sigma-Aldrich (St. Louis, MO, USA). Scotchbond™ Universal Adhesive, adhesive micro brush applicator and Filtek™ Supreme XTE composite universal restorative material was purchased from 3 M ESPE, St Paul, MN, USA.

### Fabrication of nanoparticles

#### Fabrication of BLG-NCA

A suspension of 20 mL of ethyl acetate mixed with 1.2 g of triphosgene (4.2 mmol) and 2.0 g of _L_-glutamic acid γ-benzyl ester (BLG) (8.4 mmol) was prepared, respectively. Both mixtures were stirred for 2 h at 75 °C to achieve a clear solution that was later evaporated under reduced pressure and crystallized three times using ethyl acetate and n-hexane. This mixture was subsequently dried at room temperature under vacuum and yielded a total of 1.7 g (80%) of BLG-NCA.

#### Preparation of MSN and MSN-APS

Nanoparticles were prepared according to the procedure as previously described [12]. In this study, MSN-PGA/CHX at two different concentrations of CHX (25 and 50 mg) were fabricated. As a control, MSN-PGA-Blank and MSN-CHX (25 and 50 mg) were fabricated. CTAB (1 g) was added into a solution containing 400 mL of distilled water and 2.5 mL of 2 N NaOH and vigorously stirred at 75 °C for 3 h. Later, 2.5 mL of TEOS was rapidly added into the solution and incubated for 2 h. The subsequent white precipitate was filtered and rinsed with ethanol and dried overnight under vacuum at 40 °C that produced a white powder (MSN-CTAB). MSN-CTAB (0.1 g) was dispersed in 20 mL of ethanol and heated to 85 °C. To functionalize MSN with glutamic acid, 0.1 mL APS was added into the dispersion. The obtained mixture was centrifuged, washed again with ethanol several times, and dried overnight under vacuum at 45 °C for 12 h to produce another white powder of MSN-CTAB-APS. CTAB was separated from MSN-CTAB and MSN-CTAB-APS by refluxing in an ethanol solution of ammonium nitrate (NH4NO3/C2H5OH, 10 mg/mL) for 6 h at 80 °C. The CTAB-removed product was extracted and dried to produce MSN and MSN-APS as a white powder, respectively.

#### Synthesis of poly(γ-benzyl-_L_-glutamate) grafted over MSN (MSN-PBLG)

Synthesized BLG-NCA of 0.8 g was added in 10 mL of dry DMF and mixed with a solution of 0.1 g MSN-APS in 20 mL of dry DMF. Continuous stirring of the mixture was carried out for 3 days at 40 °C. The mixture was centrifuged, washed with ethanol, and dried overnight under vacuum for 12 h at 45 °C, to form a white powder of MSN-PBLG.

#### Preparation of MSN-PGA and chlorhexidine loading

**Fig. 1:**
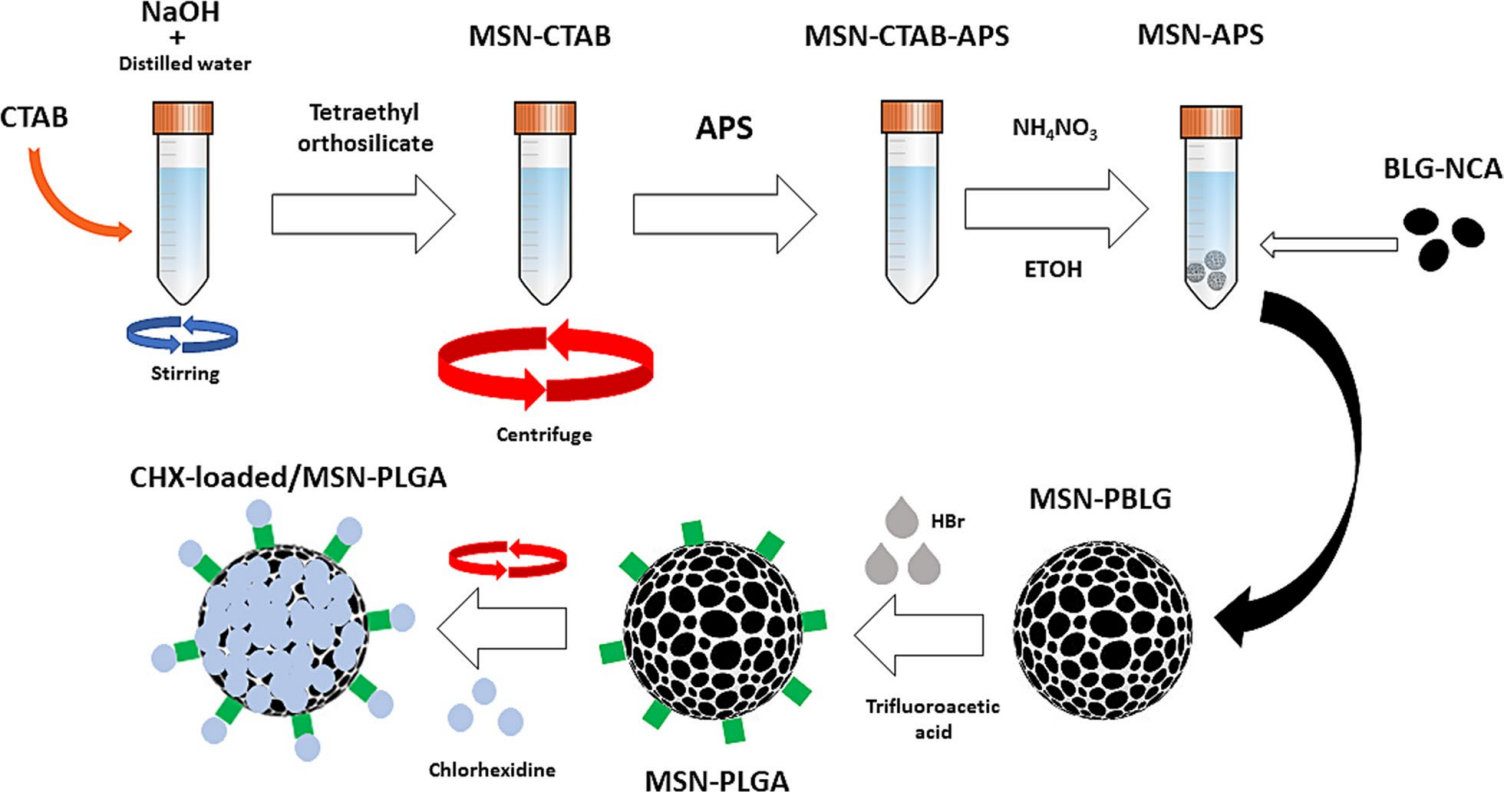
Schematic diagram showing fabrication technique of CHX-loaded MSN-PLGA

MSN-PBLG of 0.1 g was dispersed in 10 mL trifluoroacetic acid (TFA) in an ice bath and HBr (1 mL, 33 wt% in acetic acid) was added dropwise within 10 min and stirred for 2 h. The reaction mixture was poured into 40 mL ice-cold dry ether, followed by centrifugation, washing with distilled water, and drying over-night under vacuum at 45 °C for 12 h, to form a white powder of MSN-PGA. The incorporation of CHX into MSN and MSN-PGA was adopted and modified from previous protocols [11, 23]. In brief, 25 and 50 mg of CHX was dissolved in 5 mL of dichloromethane added with 50 mg of MSN or MSN-PGA, respectively, and sonicated for 12 min and slowly shaken at room temperature for 24 h, followed by centrifugation and vacuum drying (Fig. 1).

### Percentage of drug (CHX) encapsulation/loading

The obtained free CHX was separated from encapsulated CHX by centrifuging the mixture at 25 °C at 10,000 rpm for 10 min. The supernatant was conserved for evaluating the drug loading content. The quantity of free CHX was estimated using a spectrophotometer (UV-1900i UV–VIS, Shimadzu, Japan) at 289 nm wavelength. All sampling with their measurements were performed in triplicate. The drug encapsulation-efficiency and drug loading were calculated using the respective formulas:

$${\text{Percentage of drug loading}} = {\text{Weight of CHX }}/{\text{ Weight of CHX}} - {\text{loaded}}/{\text{MSN}} - {\text{PGA}} \times {1}00\%$$

$${\text{Percentage of drug encapsulation}} - {\text{efficiency}} = {\text{ Weight of CHX }}/{\text{ Weight of CHX used for encapsulation}} \times {1}00\%$$

Nanoparticles were synthesized where CHX was loaded within MSN and PGA to produce five different types of groups according to the percentage of the CHX as:

MSN-PGA/Blank; 25:50 CHX-loaded/MSN; 50:50 CHX-loaded/MSN; 25:50:50 CHX-loaded/MSN-PGA and 50:50:50 CHX-loaded/MSN-PGA.

### Dynamic light scattering

The control (MSN-PGA/Blank) and experimental nanoparticles (25:50 and 50:50 CHX-loaded/MSN and CHX-loaded/MSN-PGA at ratios of 25:50:50 and 50:50:50) were subjected to dynamic light scattering (Malvern Mastersizer Nano ZS, UK) for determination of *z*-average particle-diameter, zeta-potential, and size-distribution. Nanoparticles diluted in distilled water (1:100 wt/v) were analyzed at 37 °C with a scattering angle of 90° (n = 10/group). All sampling with their measurements were performed in triplicate.

### Morphological characterization of the nanoparticles

The morphological features of all types of nanoparticles were examined under scanning electron (Verios, XHR SEM) and transmission electron microscope (TEM, FEI Titan G2 80–200 Tokyo, Japan) coupled to energy-dispersive X-ray spectroscopy (EDS—Oxford Instruments AZtecEnergy software, USA) for elemental analysis. Nanoparticles were cleaned using absolute ethanol for any surfactants and sonicated for 5 min. A droplet of aqueous particle dispersion was allowed to evaporate on a round carbon-coated copper mesh grid (Emgrid, Australia) stabilized with the help of Dumont tweezer (ProSciTech, Australia). The samples were imaged at 200 kV under the TEM.

### pH directed chlorhexidine release from the nanoparticles

To study the *in-vitro* CHX release, nanoparticles were suspended in 5 mL of PBS solution at room temperature with constant slow stirring. To emulate different biological environments, two PBS solutions with two different pH values: 7.4 and 5.0 were investigated up to 24 h. The volume of the solution was kept constant by collecting 3 ml of the solution and at the same time replacing it with 3 mL of fresh PBS at appropriate time intervals. Subsequently, the specimens were centrifuged and the percentage of released CHX was measured using a spectrophotometer (UV-1900i UV–VIS, Shimadzu, Japan) at 289 nm wavelength. All sampling with their measurements were performed in triplicate.

### Spectral analysis of the nanoparticles

Raman signatures were recorded using Raman spectroscopy (WITec Alpha300 + , GmbH, Ulm, Germany) to confirm the inclusion of CHX into the nanoparticles. The instrument was calibrated using a silicon wafer at a magnification of 20 × 0.5. Nanoparticles were fixed on the transparent glass slide (spread dimensions: 0.8 cm width and 1 cm length) and placed on the microscope sample stage. After choosing a 100 × 0.5 objective lens and focusing the sample surface, an optical image was taken of the sample area. All Raman analyses were performed after calibration by selecting 50-micron fiber 532 nm laser. The fitting of the Raman spectrum was done using a Voigt line shape function via non-linear least squares (GRAMS32 AI Version 6.00 Peak Fit). The Raman images acquired were transferred into CytoSpec software where the entire data between 400 and 3200 cm^−1^ were pre-processed and normalized in addition to the removal of cosmic rays. Twenty spectra were recorded per specimen to establish reproducibility of the wavelength of at least ± 1.5 cm^−1^. The resolution within the order of the system was averaged to 5 cm^−1^ while keeping the spectrometer slit at 100 µm width measuring the Raman at 520 cm^−1^ of the silicon spectrum.

To further confirm the spectral characteristics, Fourier transformed infrared (FTIR) analysis using attenuated total reflection (ATR) was used with the spectrum range between 400 and 4000 cm^−1^ at a resolution of 4 cm^−1^. For the analysis, 10 mg of nanoparticles from each group was placed onto the diamond crystal on a potassium bromide slab. The powder was pressed against the diamond crystal with a flat pressure anvil connected to a pressure device and spectrum were recorded for all the powders. Each sample was run in triplicate to view if there was any difference recorded.

### Biofilm characterization and MTT assay

The MTT solution totaling 0.5 mg/ml from 3-(4,5-Dimethylthiazol-2-yl)-2,5-diphenyltetrazolium bromide kit] (MTT, Sigma-Aldrich, St. Louis, MO, USA) was prepared. The bacterial suspensions of *Streptococcus mutans* (*S. mutans*) (ATCC UA159) were prepared from the inoculum of overnight cultures and adjusted to OD_600_ of 0.5McFarland turbidity (∼10^8^ bacteria/mL). *S. mutans* were incubated anaerobically for 24 h at 37 °C in Brain–Heart infusion (BHI) and suspensions adjusted to 1 × 10^8^ CFU/ml. All suspensions were transferred into 24-well plates. Later, 10 µL of the bacterial suspension was transferred into each well containing 2 mL of BHI and 1% sucrose and incubated for 24 h at 37 °C. The non-adherent bacterial cells were washed away by PBS solution. For antibacterial evaluation, sterile filter paper-disks impregnated with 25 µL of MSN-PGA, 25:50 CHX-loaded/MSN, 50:50 CHX-loaded/MSN, 25:50:50 CHX-loaded/MSN-PGA, and 50:50:50 CHX-loaded/MSN-PGA nanoparticles (*n* = 9), were placed inside 12-well plates followed by incorporation of 2 mL of MTT solution and incubated at 37 °C for 24 h. The MTT solution was pipetted out and exchanged with 2 mL of dimethyl sulfoxide (DMSO). The well-plates were gently shaken for 15 min and the absorbance read in a spectrophotometer (UV-1900i UV–VIS, Shimadzu, Japan) at 560 nm wavelength.

### Cytotoxicity evaluation

The cytotoxicity of the nanoparticles was investigated as described in a previous protocol (n = 9) [23] using dental pulp stem cells (DPSCs) (Alameda, California, USA). Cells were seeded at 1 × 10^4^cells/well (passage 4) in a 96-well plate, incubated overnight and exposed to 25, 50 and 75 µg/mL of MSN-PGA, 25:50 CHX-loaded/MSN, 50:50 CHX-loaded/MSN, 25:50:50 CHX-loaded/MSN-PGA, and 50:50:50 CHX-loaded/MSN-PGA nanoparticles for 24, 48 and 72 h (*n* = 7/group at each concentration and incubation time). Untreated DPSCs without any treatment were used as control. The MTS [(3-(4,5-dimethylthiazol-2-yl)-5-(3-carboxymethoxyphenyl)-2-(4-sulfophenyl)-2H-tetrazolium)] reagent (CellTiter 96 AQueous One Solution Assay, Promega, USA) prepared in DMEM was added to each well followed by a 2 h incubation in 37◦C with 5% CO_2_. The microplates were read at 560 nm in a spectrophotometer (UV-1900i: UV–Vis Shimadzu, Kyoto, Japan) and the cell viability (expressed in percentage) was assessed after 0, 24, 48, and 72 h [24]. All tests were performed in triplicates.

### Delivery of nanoparticles to demineralized dentin specimens

Sound human molars (21–35 years) were used for investigating the delivery of nanoparticles to demineralized dentin substrates through micron-sized dentinal-tubules. Following extraction, teeth were stored in 0.2% sodium azide at 4 °C to inhibit microbial growth and were used within 2 months from the time of extraction. Dentin specimens were prepared for nanoparticle treatment as described previously [25]. Following preparation, specimens were randomly grouped to be treated with MSN-PGA, CHX-loaded/MSN and CHX-loaded/MSN-PGA (at CHX/MSN-PGA ratios of 25:50:50 and 50:50:50) carried on distilled water at a nanoparticles/carrier ratio of 2/1 (*w/v*) for further investigations. The exposed outer dentin-surfaces were etched with 35% phosphoric acid gel for 15 s, rinsed with distilled water for15 s, and dried with an air-syringe for 2 s leaving the dentin surface slightly moist. A drop-wise application of 25 µL of nanoparticles/carrier suspension to each dentin specimen for 60 s was followed by surface rubbing for 5 s with a micro brush applicator. Following nanoparticles application, the dentin surface was left undisturbed for 15 s, gently air-blown for 3 s, and blot-dried by an absorbent paper to remove excess water. The dentin specimens were then prepared for SEM examination (Zeiss 1555 VP-FESEM, Japan) and resin/dentin bonding procedure.

### Resin/Dentin bonding and SEM investigation

Following nanoparticles delivery to demineralized dentin-substrates, a two-step etch-and-rinse dentin bonding system was applied according to the manufacturer's instructions. Subsequently, each tooth specimen was restored to a 4-mm resin composite restoration in equal increments with each increment light-cured for 20 s (Curing Light 2500; 3 M ESPE, MN, USA). The restored teeth specimens were stored in distilled water for 24 h at 37 °C to accelerate polymerization reaction followed by occluso-gingival sectioning into 1 mm slabs using a low-speed diamond saw under running water. Each obtained resin-dentin slab was polished with increasingly fine diamond pastes (3 µm and 1 µm) and cleaned ultrasonically for 10 min. Further, the slabs were air-dried for 48 h, gold sputter-coated, and viewed by FESEM (Zeiss 1555 VP-FESEM, Japan) and for respective EDX analysis (EDS—Oxford Instruments AZtecEnergy software, USA).

### Micro-tensile bond strength

Microtensile bond testing was originally designed to allow the assessment of bond strengths between dental adhesive materials and regions of tooth tissue. Laboratories use micro-tensile bond strength (µ-TBS) testing to compare products or assess the influence of experimental parameters on adhesive-tooth bond strength. In our study, we tested the µ-TBS using 5 *wt*.% of all the nanoparticles separately added in the commercial adhesive (Scotchbond™ bond, 3 M ESPE, USA) [11]. The restored teeth were sectioned using a low-speed diamond saw (Buehler, Lake Bluff, IL, USA) under water coolant into resin–dentin beams (0.9 × 0.9 mm) and stored in artificial saliva (pH 7.4) for one week. The artificial saliva was used as the testing medium and prepared according to the protocol described by Levallois et al. [26] that involves the dissolving of reagents (0.125 M NaCl, 0.964 M KCl, 0.189 M KSCN, 0.655 M KH_2_PO_4_, 0.2 M Urea, 0.229 M CaCl_2_ 2H_2_O, 0.76 M Na_2_SO_4_ 10H_2_O, 0.178 M NH_4_Cl and 0.631 M NaHCO_3_) in distilled water (pH = 7.4) to produce a total volume of 1.0 L. The beams in each group (*n* = 75) were then randomly divided into 5 subgroups (*n* = 15 in each subgroup). The samples were tested for µ-TBS immediately following the one-week and 9 months storage in the artificial saliva (pH 7.4). The artificial saliva solution was replenished every 7 days and the pH was re-checked using a pH meter (Orion 818 pH meter, Thermo Fisher Scientific, USA). For bond strength testing, each beam was mounted on a metallic jig fixed to a universal testing machine (Instron E3000, Microtester, Instron Corp., Canton, MA) using cyanoacrylate adhesive (Zapit; Dental Ventures of America, Corona, CA, USA). A tensile load was applied at a crosshead-speed of 0.5 mm/min^−1^ until failure. For determining µTBS in MPa, the de-bonded beams were removed, and the cross-sectional area was measured at the site of fracture to the nearest 0.01 mm with the help of a digital caliper (Model 500-196-20, Mitutoyo Digimatic Caliper).

### MMP-8 and Cathepsin profilometry

The purpose of estimating MMP-8 and cathepsin profiles from the teeth samples after application of CHX-loaded/MSN-PGA was to identify the inhibition role of CHX towards endogenous MMP-8 and cysteine cathepsin. The formulated adhesive with the addition of CHX is required in a way to inhibit the degradation of tooth collagen by oral bacteria. For testing these assays, the dentin was cut from the extracted teeth (n = 16) and segments pulverized using liquid nitrogen to obtain powder using a mortar and pestle (Reimiller, Reggio Emilia, Italy). Five 1 g aliquots of dentin powder were demineralized with 10wt% H_3_PO_4_ for 24 h at 27 °C and later thoroughly rinsed in deionized water with constant stirring at 4 °C for 1 h. The groups were further treated with all nanoparticles by introducing a slurry made from PBS (0.1 mM). The dentin powder was suspended in an extraction buffer for 24 h to extract the proteases. Supernatants were collected after centrifugation at 25,000 rpm for 25 min at room temperature. The supernatants were dialyzed in bags with 30-kDa molecular cut-off overnight, lyophilized and frozen at − 20 °C until they were analyzed for MMP-8 and cysteine cathepsin (CTX) using enzyme-linked immunosorbent assay (ELISA) (Human MMP-8 ELISA Kit—Lot #5619 for MMP-8; Human CTSK/Cathepsin K ELISA Kit—Lot #5614 for cathepsin K, both from Lifespan Biosciences, Seattle, WA, USA) according to manufacturer’s instructions.

### Statistical analysis

Data were presented in means and their standard deviations. Normality testing was performed before running any statistical test. Specialized statistical software was used to analyze data using one-way ANOVA followed by Tukey–Kramer *posthoc* test. The significance level was set at p < 0.05.

## Results

### Characterization of the fabricated nanoparticles

**Fig. 2:**
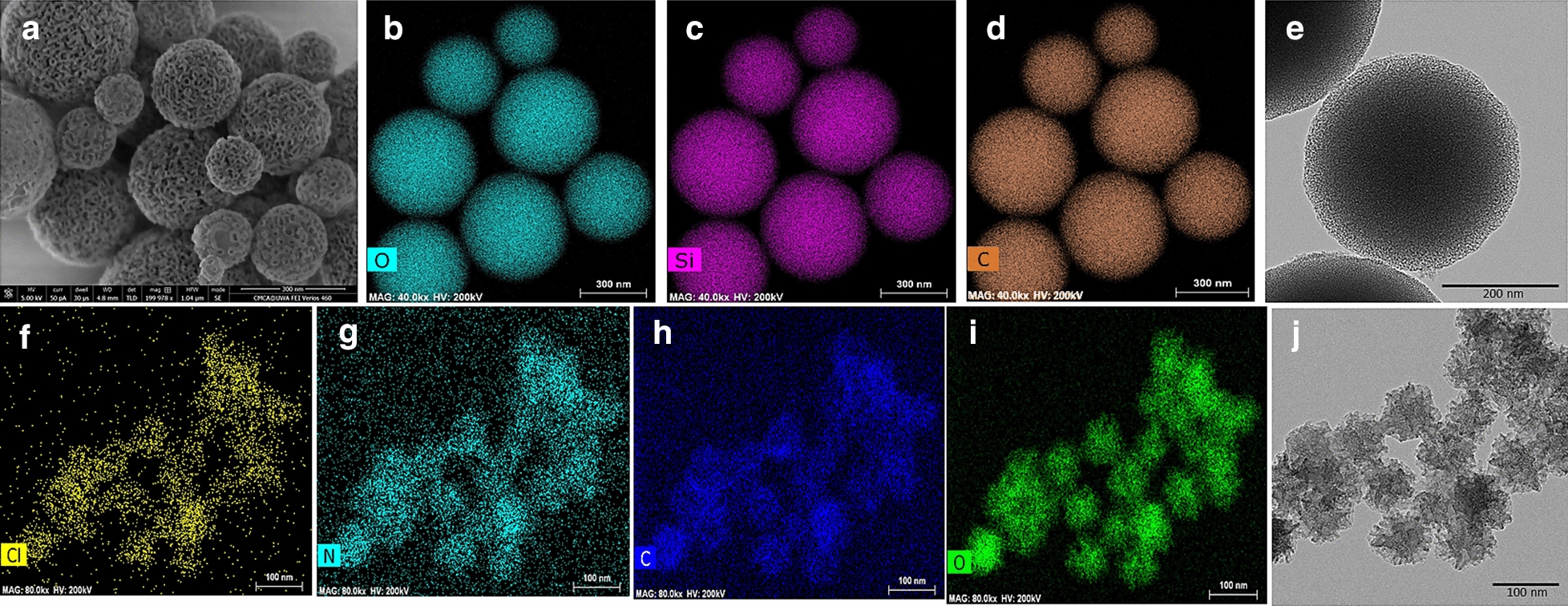
**a** Selected SEM and **e** TEM image for CHX-loaded/MSN. Associated TEM-EDS elemental analysis confirming the presence of **b** oxygen, **c** silica, and **d** carbon for CHX-loaded/MSN. **f**–**i** Associated EDS elemental analysis and **j** TEM image of the CHX-loaded/MSN-PLGA nanoparticles. The Carbon (**h**) is associated with PLGA modification and chlorine (**f**) and nitrogen (**g**) from chlorhexidine. CHX-loaded/MSN nanoparticles exhibited a more uniform morphology with relatively larger particle size compared to CHX-loaded/MSN-PLGA which showed more irregular particle morphology

Representative SEM and TEM micrographs with the associated EDX mapping of the nanoparticles is demonstrated in Fig. 2. CHX-loaded/MSN nanoparticles exhibited a more uniform morphology with a relatively larger particle size compared to CHX-loaded/MSN-PGA which showed more irregular particle morphology. EDX analysis revealed the existence of silicon, oxygen, chlorine, and nitrogen in all CHX-loaded nanoparticles. Carbon was associated with PGA modification of MSN (CHX/MSN-PGA).

Table 1 shows the results of the physicochemical characteristics of the nanoparticles. The unloaded MSN-PGA/Blank nanoparticles were ∼107 nm in diameter with a size larger than CHX-loaded/MSN-PGA nanoparticles. A significant reduction in *z*-average diameter from ∼98 to ∼84 nm was exhibited upon increasing the CHX content of MSN nanoparticles. The z-average diameter reduced further in CHX-loaded/MSN-PGA compared to CHX-loaded/MSN. Similarly, a decrease in particle size also showed a decrease in PDI values, suggesting an acceptable homogeneity level for all fabricated nanoparticles. An overall negative charge of − 15.42 mV was recorded for MSN-PGA/Blank nanoparticles, whereas CHX inclusion in MSN significantly shifted the surface charge to positive values ranging from 23.67 to 29.51 mV. The CHX-loaded/MSN-PGA demonstrated an even higher positive surface charge compared to CHX loaded/MSN.

**Table 1:** Mean and standard deviation of the percentage of drug encapsulation efficiency (DEE), drug loading (DL), nanoparticle recovery, nanoparticle size (z-average diameter), zeta potential (ζ) and polydispersity index of MSN-PLGA/Blank, CHX-loaded/MSN and CHX-loaded/MSN-PLGA at 25 and 50 mg of CHX incorporation, respectively

Groups	DEE (%)	DL (%)	Nanoparticle recovery (%)	z-Average diameter (nm)	Zeta potential (mV)	Polydispersity index
MSN-PLGA/Blank	–	–	57.1 ± 11.6^A^	107.4 ± 7.4^A^	− 15.4 ± 1.9^A^	0.27 ± 0.02^A^
25:50 CHX/MSN	81.4 ± 5.9^A^	15.3 ± 2.3^A^	65.5 ± 13.9^B^	97.9 ± 4.1^B^	23.6 ± 9.9^B^	0.18 ± 0.04^B^
50:50 CHX/MSN	85.4 ± 3.7^A^	19.7 ± 1.9^AB^	66.8 ± 12.8^B^	84.2 ± 5.9^C^	29.5 ± 9.4^B^	0.15 ± 0.01^B^
25:50:50 CHX/MSN-PLGA	92.7 ± 6.6^B^	20.4 ± 2.5^B^	67.6 ± 11.4^B^	81.2 ± 3.4^C^	41.3 ± 8.1^C^	0.083 ± 0.01^C^
50:50:50 CHX/MSN-PLGA	95.8 ± 5.6^C^	24.1 ± 3.1^B^	64.1 ± 15.2^B^	74.9 ± 4.6^D^	42.9 ± 6.7^C^	0.065 ± 0.01^D^

Dissimilar uppercase letters show statistical significance at p < 0.05 within each column

### Chlorhexidine content

**Fig. 3:**
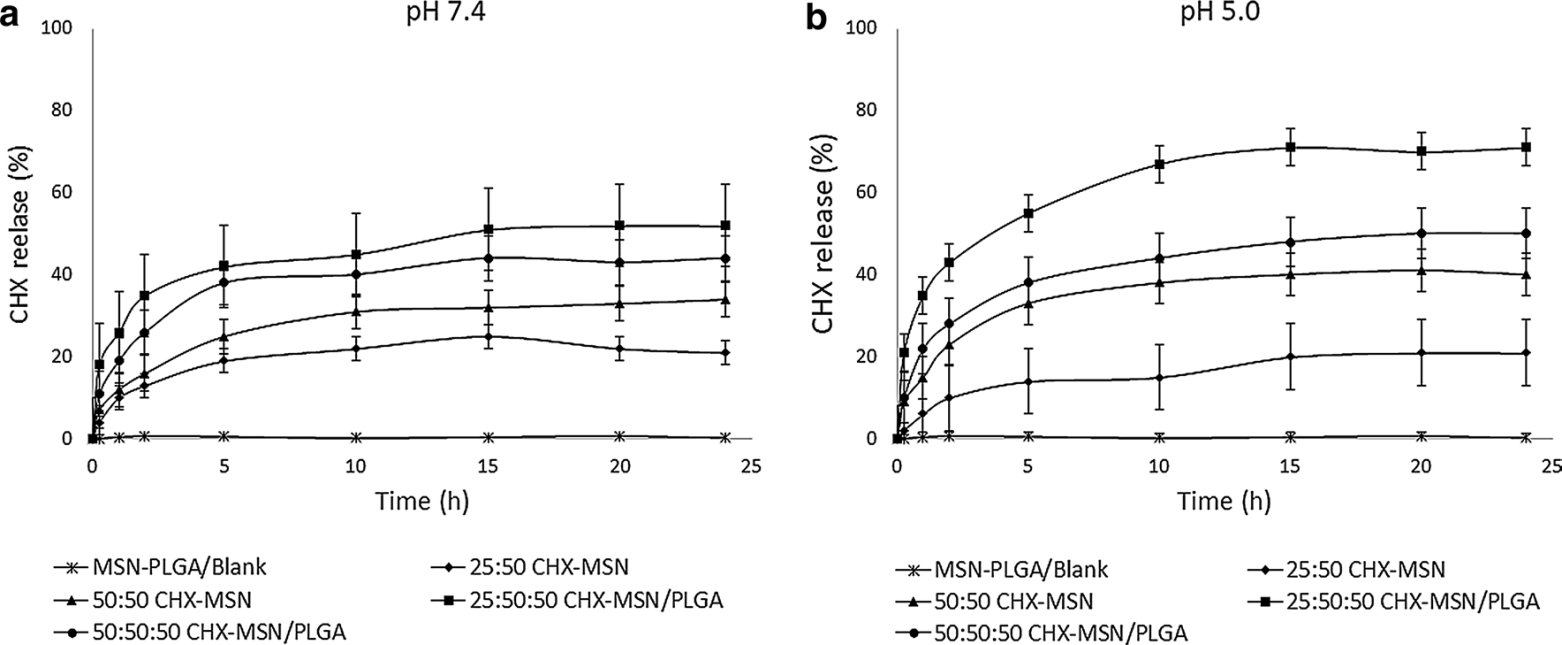
In-vitro CHX release profiles of 25:50 CHX-loaded/MSN, 50:50 CHX-loaded/MSN, 25:50:50 CHX-loaded/MSN-PLGA, 50:50:50 CHX-loaded/MSN-PLGA and MSN-PLGA/Blank nanoparticles at pH **a** 7.4 and **b** 5.0 up to 24 h

The drug encapsulation and loading of CHX-loaded/MSN, MSN-PGA/Blank, and CHX-loaded/MSN-PGA are reported in Table 1. It is observed that an increase in the percentage of drug encapsulation efficiency from 81 to 85% in CHX loaded/MSN and 92–95% in CHX loaded/MSN-PGA proportionately increased with increasing the amount of CHX during the fabrication of nanoparticles. Therefore, encapsulation efficiency of 81% produced a drug loading of 15%, substantially increasing to 19% upon further adding of CHX to 50 mg. The trend was even significantly higher upon grafting PGA over MSN with drug encapsulation efficiency of 96% that produced a drug loading of 24%. The in vitro drug release behaviors of MSN-PGA/Blank, CHX-loaded/MSN, and CHX-loaded/MSN-PGA nanoparticles at pH 7.4 and 5.0 were experimented and shown in Fig. 3a, b. The drug release rate of CHX-loaded/MSN-PGA was more pH-dependent and increased with the decrease of pH. The cumulative release of CHX amount from CHX-loaded/MSN-PGA reached up to 70% after 24 h at pH 5.0, much higher than that at pH 7.4, which was 49% only. At lower pH, the trend of higher drug release was seen in 25:50:50 CHX-loaded/MSN-PGA. Nanoparticles 50:50:50 CHX-loaded/MSN-PGA and 50:50 CHX-loaded/MSN showed a close pattern; however, 50:50:50 CHX-loaded/MSN-PGA showed slightly higher drug release then 50:50 CHX-loaded/MSN that indicated the drug release was similar and the difference in release amount between different pH values was not significant. The drug release of CHX-loaded/MSN was also slightly pH-dependent. However, CHX-loaded/MSN-PGA exhibited a more significantly pH-dependent drug release behavior than CHX-loaded/MSN.

### Spectral analysis

**Fig. 4:**
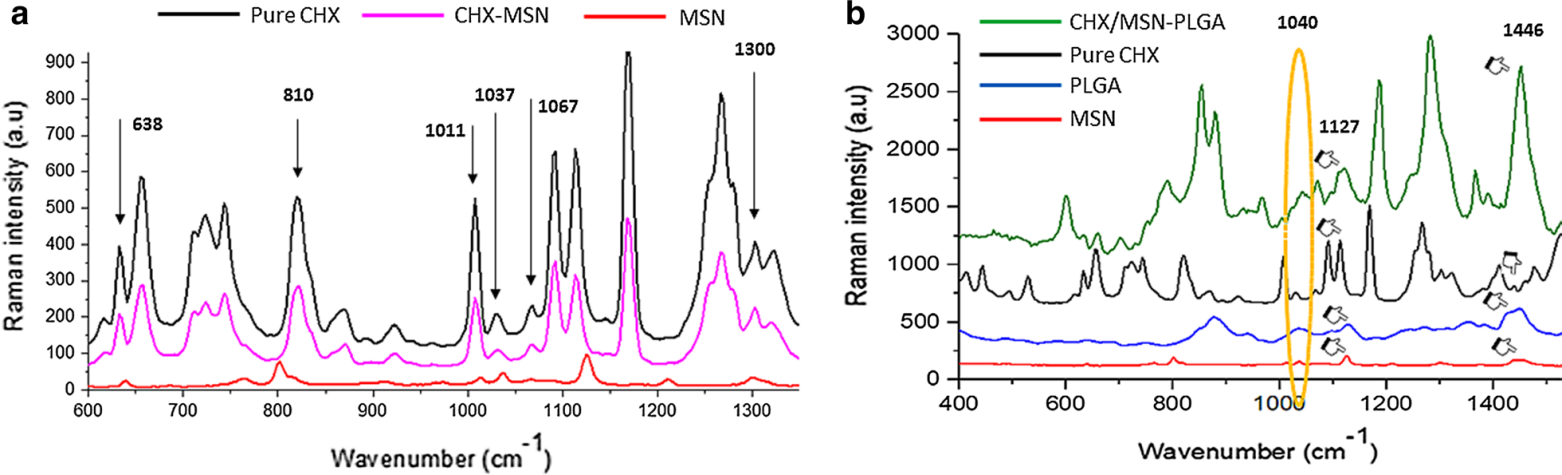
Raman spectra of all synthesized nanoparticles MSN, PLGA and pure chlorhexidine powder are shown (**a**) When using excitation wavelength of 532 nm laser, the synthesized mesoporous silica powder (red spectrum) shows a Raman spectrum having prominent signals at 638, 802 and 1300 cm^−1^. The band around 1300 is attributed to the lactic units. The peaks between 400 to 1350 cm^−1^ are assigned to pure CHX powder. CHX-loaded/MSN exhibited a characteristic peak of Raman scattering at 638, 802, 1011, 1035, 1067 and 1300 cm^−1^. **b** The Raman spectrum at 1446 cm^−1^ for CHX-loaded/MSN-PLGA is suggestive of anti-symmetric vibration from the lactic unit corresponded as CH_2_ deformation which is confined to a lower intensity in CHX-loaded/MSN groups. The assignments at 1040 and 1127 cm^−1^ corresponds to CH_3_ rocking and CH_2_ wagging

Raman spectra of all synthesized nanoparticles MSN, PGA, and pure chlorhexidine powder are shown in Fig. 4a, b. When using an excitation wavelength of 532 nm laser, the synthesized mesoporous silica powder (red spectrum) shows a Raman spectrum having prominent signals at 638, 802, and 1300 cm^−1^ [27]. The band around 1300 is attributed to the lactic units. The peaks between 400 to 1350 cm^−1^ are assigned to pure CHX powder. CHX-loaded/MSN exhibited a characteristic peak of Raman scattering at 638, 802, 1011, 1035, 1067 and 1300 cm^−1^ (Fig. 4a). The Raman spectrum at 1446 cm^−1^ for CHX-loaded/MSN-PGA is suggestive of anti-symmetric vibration from the lactic unit corresponded to CH_2_ deformation which is confined to a lower intensity in CHX-loaded/MSN groups. The assignments at 1040 and 1127 cm^−1^ corresponds to CH_3_ rocking and CH_2_ wagging [28] (Fig. 4b).

**Fig. 5:**
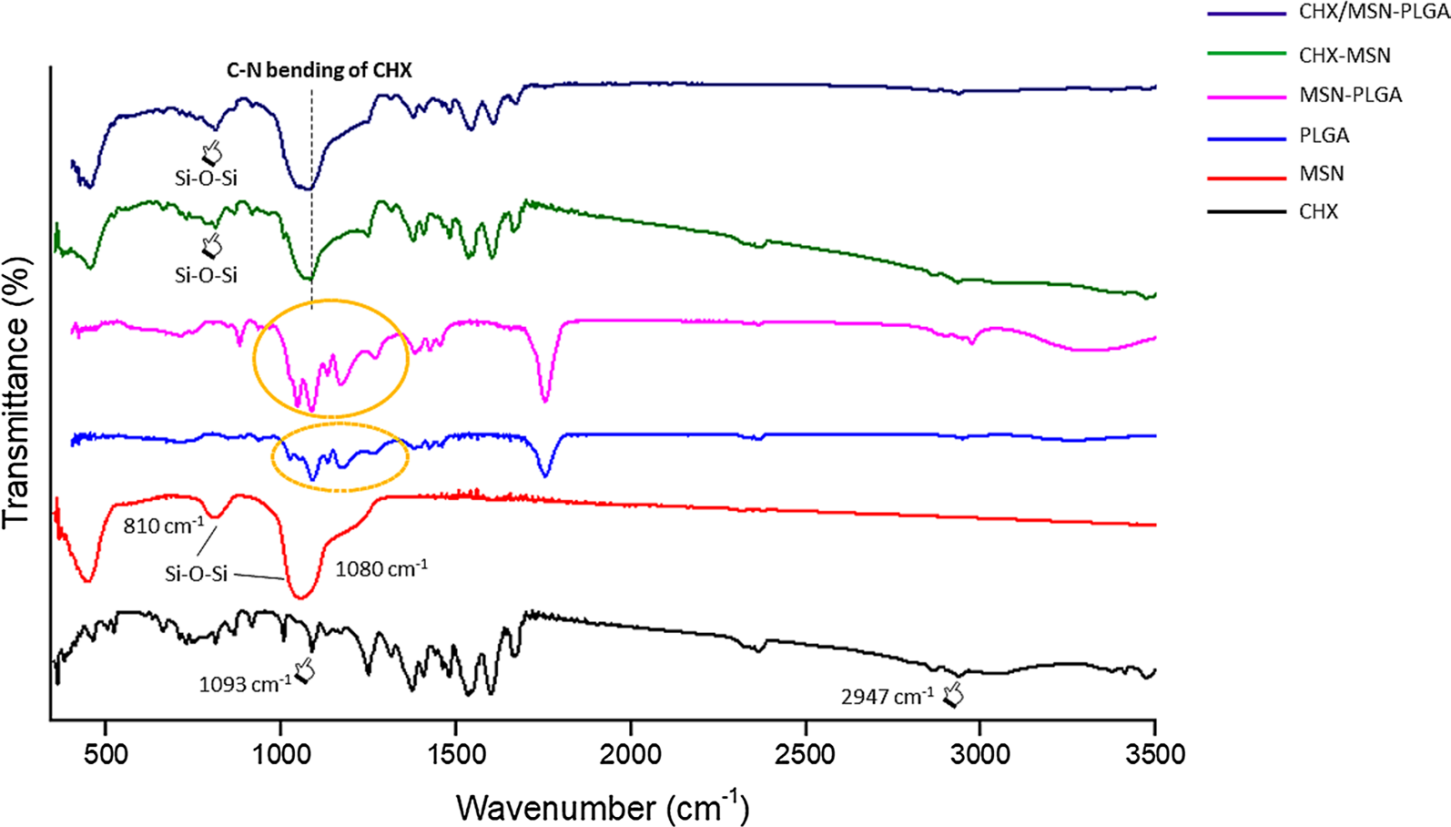
The FTIR spectra of pure CHX, synthesized MSN and PLGA has been shown for reference. Pure CHX exhibiting characteristic peaks at 1093 and 2947 cm^−1^ (pointers) that indicates C-N bending and C-H vibration. The two bands that appeared at 1080 and 810 cm^−1^ belonged to the characteristic peaks of Si–O-Si on the SiO2 framework of MSNs. PLGA displays characteristic absorption bands at 1100–1250 and 1750–1760 cm^−1^ which represent the esters and carbonyl groups (dotted circle). The above-mentioned fingerprint peaks specific to CHX can be observed in all the CHX-loaded/MSN and CHX-loaded/MSN-PLGA

The FTIR spectra of pure CHX, MSN, and PGA are shown in Fig. 5 for reference. Pure CHX exhibiting characteristic peaks at 1093 and 2947 cm^−1^ that indicates C-N bending and C-H vibration [29]. The two bands that appeared at 1080 and 810 cm^−1^ belonged to the characteristic peaks of Si–O-Si on the SiO2 framework of MSNs [30]. PGA displays characteristic absorption bands at 1100–1250 and 1750–1760 cm^−1^ which represent the esters and carbonyl groups [31]. The above-mentioned fingerprint peaks specific to CHX can be observed in all the CHX-loaded/MSN and CHX-loaded/MSN-PGA.

### Antibacterial and cytotoxicity testing

**Fig. 6:**
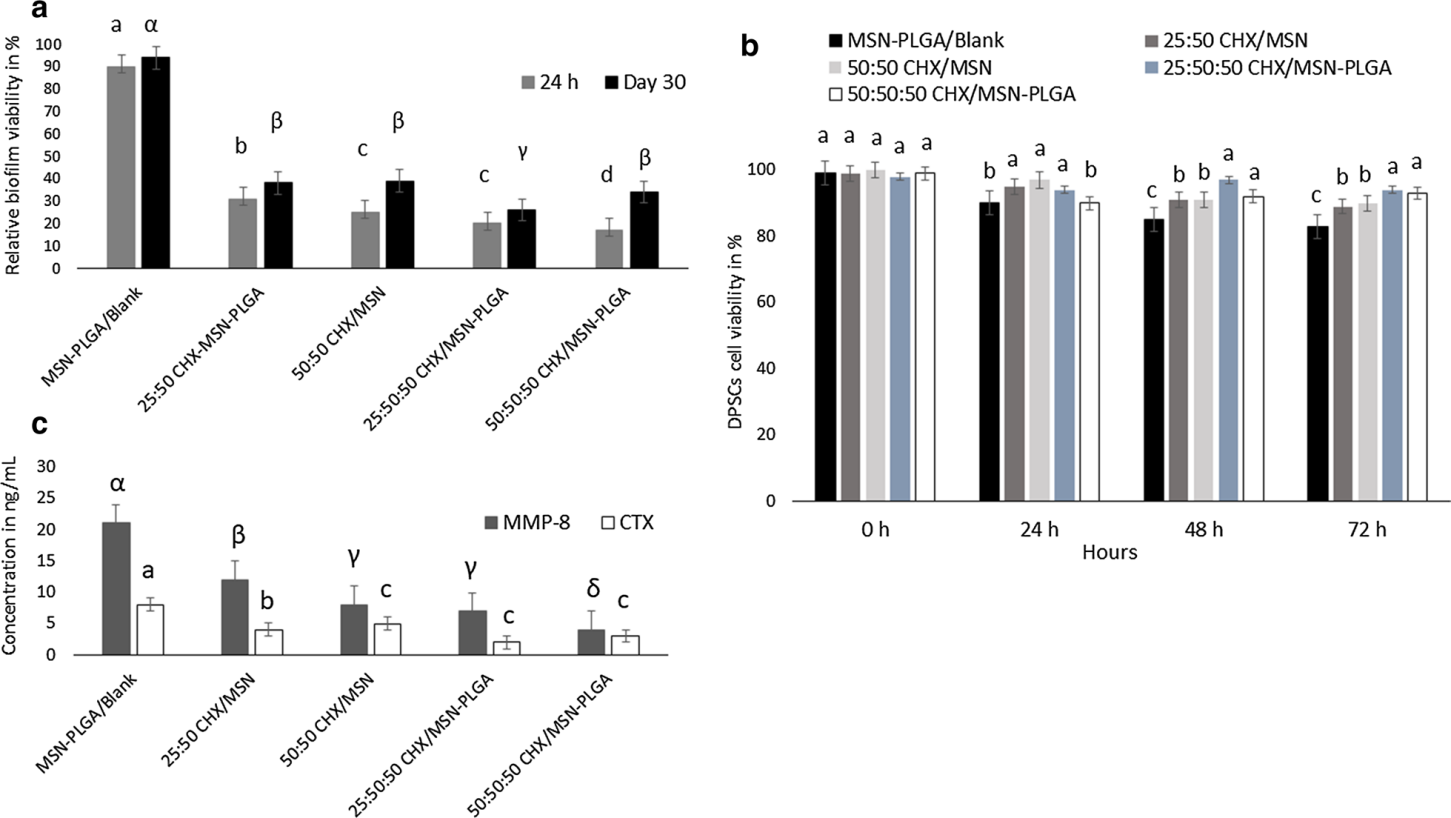
**a** MTT assay showing relative percentage of *S. mutans* biofilm viability at 24 h and 30 days; **b** percentage of DPSCs viability. **c** The results of MMP-8 and cathepsin K concentrations

Figure 6a, b shows the results of the antimicrobial activity and cytotoxicity of all the nanoparticles. For both time-periods (24 h or 30 days), the relative microbial viability significantly decreased by increasing the CHX content (*P* < 0.001). After 24 h, the 50:50 CHX-loaded/MSN and 25:50:50 CHX-loaded/MSN-PGA showed equal antimicrobial efficacies (*P* > 0.05). The highest antimicrobial capacity at 24 h was demonstrated by 50:50:50 CHX-loaded/MSN-PGA. On day 30, the bacterial viability of the group 25:50:50 CHX-loaded/MSN-PGA remained low under 30% (Fig. 6a). For cytotoxicity, the results showed significant differences in DPSCs viability between drug-loaded and unloaded nanoparticles only (*P* < 0.01) (Fig. 6b). Generally, the cell viability percentage of DPSCs exposed to MSN-PGA/Blank, CHX-loaded/MSN, and CHX-loaded/MSN-PGA, respectively was > 80% indicating low cytotoxicity profiles of experimental nanoparticles. In addition, none of the CHX concentrations showed statistical significance over time.

### µTBS

The outcomes of µTBS are summarized in Table 2. After storage in one week of neutral artificial saliva, groups containing 5 wt.% 25:50 CHX/MSN, 50:50 CHX/MSN, and 25:50:50 CHX/MSN-PGA showed no significant difference in µTBS compared with the commercial adhesive (*p* > 0.05). However, after 9 months in artificial saliva (pH 7.4) significantly highest µTBS value was recorded for 25:50 CHX/MSN and 25:50:50 CHX/MSN-PGA with no significant difference compared with the 1-week value.

**Table 2:** Mean and standard deviation of the micro-tensile bond strength (µTBS in MPa) for the modified experimental adhesives, and the commercial adhesive after one week of storage in artificial saliva and 9 months of storage in artificial saliva

Groups	One week in AS (pH 7.4)	9 months in AS (pH 7.4)
25:50 CHX/MSN	38.2 ± 5.9 A (a)	29.2 ± 7.8 A (a)
50:50 CHX/MSN	36.4 ± 7.2 A (a)	22.8 ± 6.5 B (b)
25:50:50 CHX/MSN-PLGA	39.7 ± 7.4 A (a)	28.7 ± 5.0 A (b)
50:50:50 CHX/MSN-PLGA	31.8 ± 6.3 B (a)	19.4 ± 7.6 B (b)
MSN-PLGA/Blank	32.5 ± 6.0 B (a)	18.1 ± 9.8 B (b)
Commercial adhesive (Scotchbond™ bond, 3 M ESPE)	42.3 ± 5.8 A (a)	28.5 ± 7.7 A (b)

Dissimilar uppercase letters (A–E) show statistical significance at p < 0.05 within each column

Dissimilar lowercase letters (a–c) show statistical significance at p < 0.05 within each row

### MMP-8 and cathepsin K concentrations

The results of MMP-8 and cathepsin K concentrations are depicted in Fig. 6c. The MMP-8 levels were significantly low in CHX-loaded nanoparticles as compared to unloaded-MSN-PGA (p < 0.05). The concentrations of active cathepsin-K in 25:50:50 CHX-loaded/MSN-PGA were the lowest among all groups (p < 0.05) (Fig. 6c). The PGA coating had a significant effect on the MMP-8 and cathepsin K activity.

### Characterization of nanoparticles-treated demineralized dentin substrates and adhesive-dentin interface

**Fig. 7:**
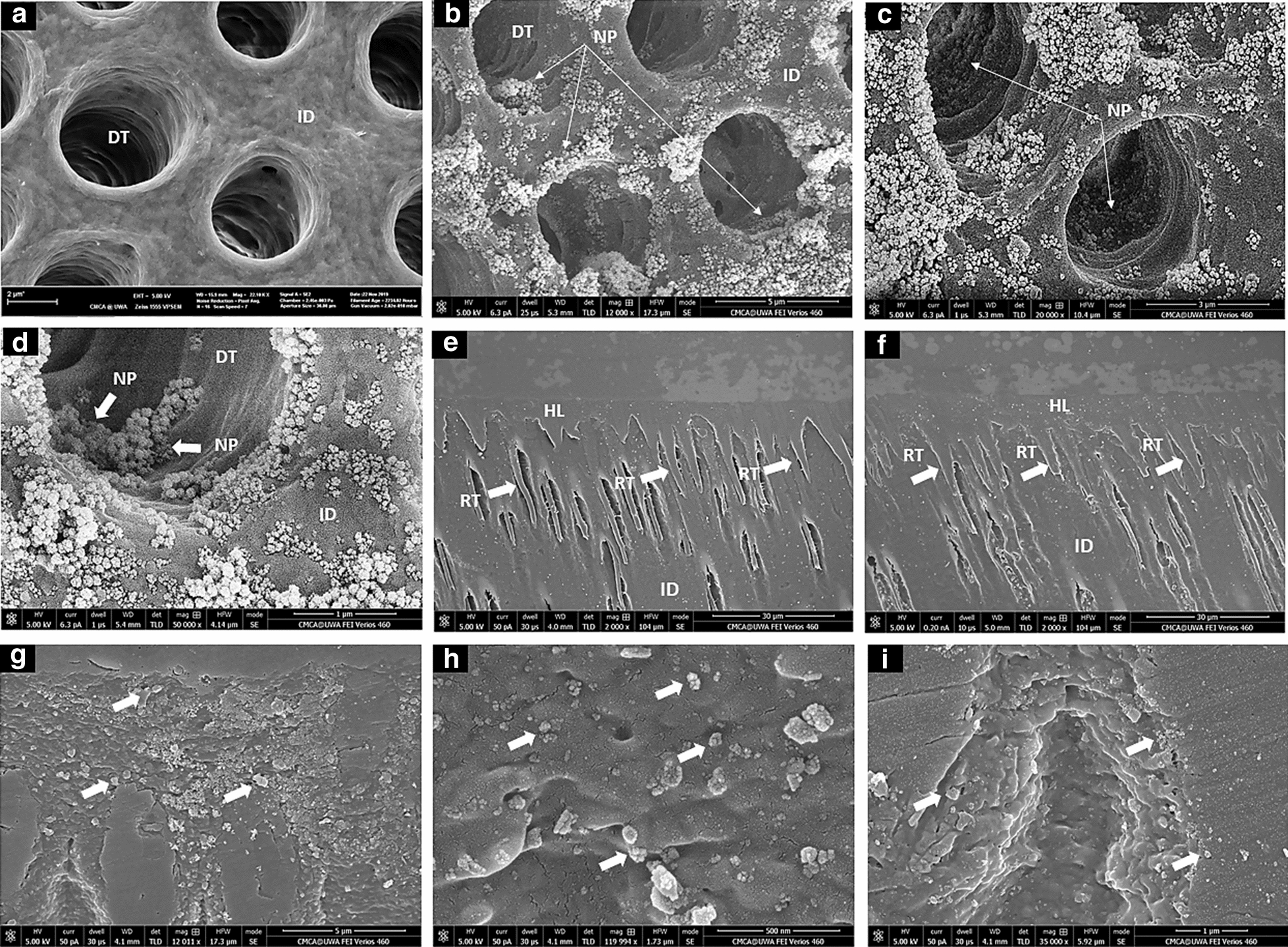
**a** Representative SEM image showing the widely open dentinal tubules after acid-demineralization. **b**–**d** Selected SEM images showing the ability of the CHX-loaded/MSN-PLGA nanoparticles (NP) delivered through the aqueous-carrier to penetrate and infiltrate inside the dentinal tubules. The nanoparticles appear to be evenly distributed, confined in dentinal tubule structure and attached to tubular wall after the air-blown and blot-drying steps. The nanoparticles are also seen to penetrate considerable depths within the dentinal tubules. **e** Representative SEM image showing the overall homogenous intact resin-dentine hybrid layer (HL) along with well-developed resin tags of the 50:50:50 CHX-loaded/MSN-PLGA and **f** 50:50 CHX-MSN nanoparticles treated dentin specimens after the application of the commercially available dentine bonding system (Scotchbond™ Universal adhesive). DT: dentinal tubules, ID: intertubular dentine, NP: nanoparticles, RT: resin tags. (**g**–**h**) Representative high magnification SEM image showing the hybrid layer with widely distributed CHX-loaded/MSN-PLGA nanoparticles (white arrowheads) and exhibiting excellent bonding. Note some areas show tight agglomerations of the nanoparticles. A higher magnification area between two resin tags showing well-penetrated nanoparticles within the deep dentinal space

The nanoparticles delivered using an aqueous-carrier showed the ability to penetrate considerable depths inside dentinal-tubules (Fig. 7). The nanoparticles appeared to be evenly distributed, confined to the dentinal-tubule structure, and attached to tubular walls after air-blowing and blot-drying steps (Fig. 7b–d). A representative overall low magnification SEM imaging of the resin-dentin interfaces of specimens treated with the 50:50:50 CHX-loaded/MSN-PGA and 50:50 CHX-MSN nanoparticles followed by the application of commercially available dentin adhesive system revealed the formation of intact resin/dentin hybrid-layer along with numerous well-developed resin-tags (Fig. 7e, f). The high magnification of dentin-bonded specimens sectioned vertically indicated a homogenous and widely distributed 50:50:50 CHX-loaded/MSN-PGA nanoparticles and exhibiting excellent bonding. Some areas of the hybrid layer showed tight agglomerations of the nanoparticles (Fig. 7g–i).

**Fig. 8:**
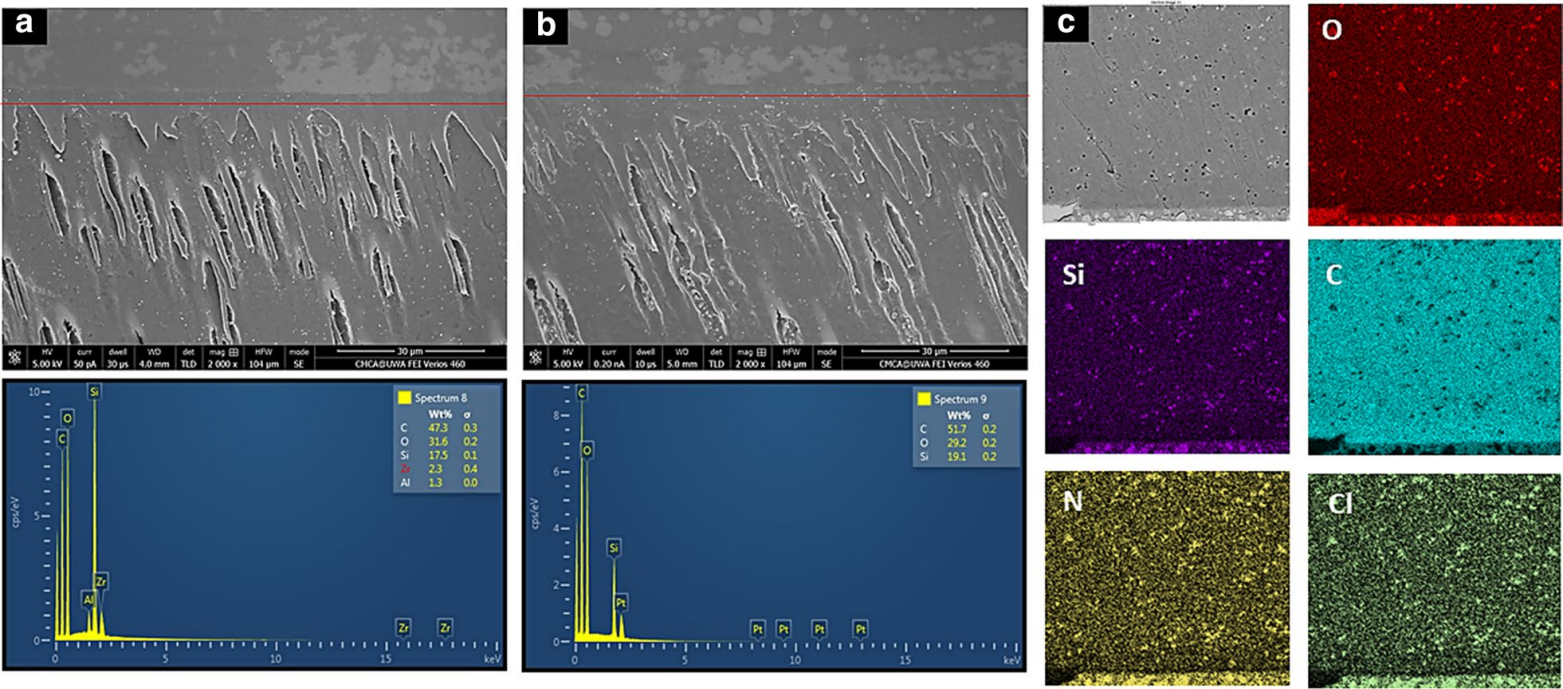
**a**, **b** Representative SEM image of resin dentin interface bonded using 50:50 CHX-MSN and 50:50:50 CHX-loaded/MSN-PLGA with their respective line EDX analysis exhibiting high percentage of amorphous carbon and silica along the line of resin-dentin interface. Note the amount of carbon increased with the grafting of PLGA over the MSN surface in 50:50:50 CHX-loaded/MSN-PLGA. **c** EDX mapping of resin matrix within hybrid layer from 50:50:50 CHX-loaded/MSN-PLGA showing nanoparticles distribution and the presence of carbon (C) and silica (Si) indicating MSN and PLGA within the resin matrix and distribution of nitrogen (N) and chlorine (Cl) as an evidence for chlorhexidine

The line EDX performed on specimens bonded with MSN-PGA/Blank and 50:50:50 CHX-loaded/MSN-PGA exhibited a high percentage of amorphous carbon and silica that represents MSN-PGA with no evidence of the drug CHX. The presence of nitrogen and chlorine depicts the presence of CHX along the line of the resin-dentin interface (Fig. 8a, b). EDX mapping of resin matrix within hybrid layer from 50:50:50 CHX-loaded/MSN-PGA showing nanoparticles distribution and the presence of carbon (C) and silica (Si) for MSN and PGA within the resin matrix and distribution of nitrogen (N) and chlorine (Cl) as evidence for CHX (Fig. 8c).

## Discussion

A complex nanocarrier system using PGA grafted over MSN demonstrating controlled release and pH-responsive behavior was formulated and investigated. For the drug release, a cationic CHX as a template drug was used. The present investigation also aimed to investigate and characterize the feasibility of CHX-loaded/MSN-PGA as nanocarriers as a potential modifier of dentin adhesives. A sol–gel technique was adopted to prepare MSN-CTAB and followed by the functionalization of amino groups on the outer surface of MSN through APS [12, 32]. Raman and FTIR analyses (Figs. 4 and 5) provided evidence for the successful preparation of these nanocarrier systems; MSN displaying strong peaks at 638, 802, and 1300 cm^−1^ while peaks between 400–1350 cm^−1^ were assigned to CHX. In addition, FTIR further confirmed the signals that appeared at 1093 cm^−1^ for CHX, 810 cm^−1^ for MSN, and peaks 1100–1250 cm^−1^ associated with CHX-loaded/MSN-PGA. The significant intensity at 810 cm^−1^ and high-frequency shift proved the nanocrystalline silicon overlapped by surrounding signals. The Raman shift to lower confinements would be best explained by the incorporation of CHX causing a change in spectral efficiency of Raman scattering (from 802 to 810 cm^−1^) which could be due to the scattering of optical photons from the silicon [33, 34]. The maximum intensities observed are inherent to the nanocrystalline silicone, a predominant fraction seen in the MSNs.

When MSN nanoparticles were examined under electron microscopy (Fig. 2), the nanoparticles appeared smooth with regular mesoporosity. The mesoporosity became irregular and less clearly defined after loading CHX signifying the adsorption of the drug onto the external surface of MSN and inside the mesoporous cavities [11]. EDS elemental analysis showed strong intra-particulate silicon, carbon, nitrogen, and chlorine in CHX-loaded/MSN-PGA nanoparticles. Studies have reported that the amount of CHX loading from CHX-loaded/MSN exceeds > 40 wt.% [23, 35], and this reasonably high drug loading complements MSN as a superior nanocarrier for the CHX. Furthermore, the loading of CHX inside MSN significantly shifted the charge from negative to positive as described in Table 1. The negative charge that MSN carries is due to the silica that undergoes dissociation of surface hydroxyl groups. The shift to the positive charge may be due to the robust electrostatic interaction and gradual diffusion of the positively charged CHX molecules [35]. This probably leads to the controlled release of the drug and therefore useful for the maintenance of bond strength of the adhesive with tooth dentin [36].

The in-vitro CHX release profiles from the nanoparticles were investigated in PBS at 7.4 and 5.0 pH values. The reason for choosing two different types of pH is because 7.4 is the normal pH of the saliva under normal physiological conditions, while 5.0 is generally the pH of the microenvironment caused by cariogenic bacteria in dental caries inside the tooth tissues [37, 38]. Therefore, to mimic the oral condition (both sound and diseased states) we chose the two pH distribution patterns for our in-vitro drug release study. Furthermore, research studies have been published that indicated a potent and stronger drug release behavior in a further decrease in pH. For instance, Jin et al. [16] studied pH-responsive mesoporous silica nanoparticles carrying ibuprofen in PBS solution with pH 7.4 and '2.0′. They reported that the drug release rate was faster at an extremely lower pH (2.0) than that at pH 7.4. In another study by Chang et al. [39], they used mesoporous silica nanoparticles as the drug-loaded nanocarrier for cancer therapy and emulate three different pH environments (7.4, 6.0, and 5.0). However, aqueous solutions of CHX salts are most stable in a pH range: 5–8; in the alkaline region (pH > 8), the CHX base is precipitated, while, in the acidic region, the preparation gradually loses its antibacterial activity because of destabilization [40]. Following this concept, the weakening of bonds at the adhesive-dentin interface has been widely studied both in vivo [41, 42] and in vitro [43–45]. Although the phenomenon remains unclear, it is suggested that microleakage and collagen degradation are primary factors that are responsible for weakening resin-dentin bonds [46]. It is proven that microleakage is caused by the residual *S. mutans* at the resin-dentin interface that maintains reduced metabolic capability and low sensitivity to antimicrobial agents [47]. Thus, it is of utmost importance to develop a drug-loaded nanocarrier system that could retain antibacterial efficacy and suppress biofilm formation at an adhesive-dentin interface. CHX has been widely experimented in several in vitro studies due to its strong antibacterial capacity and inhibition of oral biofilm formation [48, 49]. Previous studies have reported that MSN is capable of loading drugs and releasing them at considerably low pH [12, 50].

In our study, the encapsulation of CHX was relatively higher when PGA was grafted over the MSN surface. Priyadarshini and colleagues [23] stated that the percentage of encapsulation efficiency of PGA nanocarriers was proportional to increasing the amount of CHX addition. This could be attributed to the hydrophobic nature of the drug CHX that can be efficiently enclosed inside PGA nanoparticles [51, 52]. Thus, we speculate that PGA grafted over MSN would carry more CHX and show a sustained release of CHX in low pH for a long time. Along with this, the overall release of CHX from grafted PGA/MSN was higher in low pH than in high pH. MSN and PGA are two polymeric materials that demonstrate excellent controlled and pH-responsive drug delivery [53, 54]. At low pH values, the polymeric materials tend to fracture which is attributable to the crystallization of oligomeric degradation products as a consequence of low solubility of MSN and PGA [55]. Since both nanocarriers function extremely well in low pH, for this reason, these nanoparticles system after incorporating in a dentin-adhesive system would surmount the bacterial growth and show high antibacterial efficacy. The aforementioned tests in our study backed our assumptions.

Regarding the antimicrobial activity (Fig. 6a), the metabolic activity of *S. mutans* remarkably reduced by the addition of a higher percentage of CHX-loaded MSN with or without PGA even after 30 days of storage. This indicates that the antimicrobial efficiency of these groups can be maintained for a longer time. The long-term antibacterial efficacy depends significantly on the CHX release (Fig. 3a–b). All nanoparticles containing CHX showed an initial burst release, that later exhibited a constant release with similar slopes. However, the low pH created due to bacterial biofilms may trigger a higher percentage of CHX release from CHX-loaded/MSN-PGA modified adhesives enhancing the antimicrobial effect. Therefore, pH-sensitive CHX release could be of potential significance against cariogenic biofilms within dentin substrates. Various research studies document the antibacterial substantivity of CHX ranging from 48 h up to 12 weeks [56, 57]. Therefore, it is arduous to give recommendations on its clinical applicability, and further research is warranted to evaluate the antimicrobial susceptibility of CHX-loaded/MSN-PGA for clinical use. Furthermore, this study investigated *S. mutans* only which are the primary initiators of dental caries. Future studies should be conducted to test the efficacy of CHX-loaded/MSN-PLGA using multiple bacterial biofilms.

Substantial amounts of penetrated nanoparticles were revealed by SEM, verifying the capability of 60 s application of 10% nanoparticles for deep infiltration within the dentinal-tubules structure. The use of water as an experimental nanoparticles’ dispersion phase/carrier was efficient to facilitate the delivery and infiltration of Nano-PGA/CHX nanoparticles through dentinal-tubules within 60 s application-time, which is considered clinically realistic and acceptable. The ability of water to infiltrate inside slightly dried demineralized dentin-substrates carrying the dentin bonding resin is a well-known phenomenon in adhesive dentistry [58]. The close association of spherical nanoparticles across the resin-tag morphology further confirmed nanoparticle penetration, their presence inside dentinal-tubules and successful retention even after resin-infiltration. However, further advances are mandatory to estimate the nanoparticle's penetration-depth. An ethanol-containing 2-step etch-and-rinse dentin adhesive system was used to further synchronize the nanoparticles penetration-phenomena with resin-infiltration inside dentinal-tubules to form hybridized resin-tags. The prominence of air-blowing and blot-drying steps on promoting nanoparticle confinement inside dentinal-tubules is of clinical importance to conserve and protect the intact morphology of resin-dentin hybrid-layer specifically after nanoparticles degradation. However, recommendation on the use of CHX-loaded/MSN-PGA nanoparticles after acid-etching, at this point of our study, is limited to the adhesive system based on the etch-and-rinse principle. Further studies should investigate the spatial correlation of nanoparticles around and/or inside the resin tags within the structure of dentinal tubules. Moreover, to scrutinize the effect of resin infiltration on drug release characteristics of delivered nanoparticles.

With regards to the µ-TBS, it is noted that the bond strength was significantly reduced in the groups of nanoparticles containing the highest amount. Hence, it could be postulated that the u-TBS is significantly affected by the incorporation of nanoparticles and may not be affected by the drug itself.

There lies a universally stated notion that S1′ pocket of MMPs can accommodate residues and can catalytically cleaved but are not structurally open. Moreover, CHX tends to stabilize genolytic activities, and because of MMPs not being entirely free and to be attached only at the fibronectin and hemopexin sites [59]. This limitation of cleavage is speculated to have been controlled by the CHX present within the nanoparticles. This becomes a pivotal reason why the enzymatic activity is lowered as seen in specimens with a higher concentration of CHX and stable release. CHX is cationic at physiological pH and can remove calcium and zinc ions which are important for MMP activities. It also shuts down the essential cysteine and/or sulfhydryl groups on the catalytic site of the MMP enzymes [60].

This study introduced the significance of a pH-responsive CHX nanocarrier delivery system formed of MSN modified with PGA as a potential modifier of resin-based dentin adhesives in terms of improving the antimicrobial activities, proteases inhibition, and resin-dentin bonding integrity and durability. This reported pH-sensitive CHX release response could be of crucial advantage for resin-dentin bonding application due to the associated decrease in pH in the surrounding microenvironment resulting from biofilm formation, acid etching, and acidic content of bonding monomers. The concept of grafting PGA over MSN nanocarrier was only to load more CHX within the nanoparticle for high and efficient drug release. However, the preparation of PGA and grafting over MSN is a highly complex multi-step preparation that should be taken into consideration.

## Conclusion

A pH-sensitive CHX release response was noted when loaded in MSN grafted PGA nanoparticles. The formulated drug-loaded nanocarrier demonstrated excellent physicochemical, spectral, and biological characteristics. Showing considerable capacity to penetrate effectively inside dentinal tubules and having high antibacterial efficacy, this system could be potentially used in adhesive and restorative dentistry.

## Data Availability

The analyzed datasets generated during the current study are available from the corresponding author on reasonable request.

## Abbreviations

µ-TBS: Micro-tensile bond strength
ANOVA: One-way analysis of variance
APS: Aminopropyltriethoxysilane
ATR: Attenuated total reflectance
BLG-NCA: ɤ-Benzyl-L-glutamate N-carboxyanhydride
BHI: Brain–Heart infusion
CHX: Chlorhexidine
CTAB: N-cetyltri-methylammonium bromide
DPSCs: Dental pulp stem cells
EDS: Energy dispersive X-ray analysis
ELISA: Enzyme-linked immunosorbent assay
FTIR: Fourier transformed infra-red spectroscopy
MMP: Matrix metalloproteinase
MPa: Megapascal
MSNs: Mesoporous silica nanoparticles
MTS: [(3-(4,5-Dimethylthiazol-2-yl)-5-(3-carboxymethoxyphenyl)-2-(4-sulfophenyl)-2H-tetrazolium)]
MTT: 3-(4,5-Dimethylthiazol-2-yl)-2,5-diphenyltetrazolium bromide
PBS: Phosphate buffer saline
PLGA: Poly-L-glycolic acid
SEM: Scanning electron microscopy
*S. mutans*: *Streptococcus mutans*
TEM: Transmission electron microscopy
TFA: Trifluoroacetic acid
TEOS: Tetraethyl orthosilicate
